# Rethinking Arterial Spin Labeling Perfusion in Neurodegeneration Considering Global and Regional Changes

**DOI:** 10.7759/cureus.74188

**Published:** 2024-11-21

**Authors:** Emilian Kalchev

**Affiliations:** 1 Diagnostic Imaging, St. Marina University Hospital, Varna, BGR

**Keywords:** alzheimer's, arterial spin labeling, brain perfusion, dementia, mri, neurodegeneration

## Abstract

Recent advancements in arterial spin labeling (ASL) MRI have significantly improved our understanding of cerebral perfusion in neurodegenerative diseases. Traditionally, the focus has been on regional perfusion deficits corresponding to specific neural pathologies. However, this localized approach may overlook the influence of global cerebral blood flow alterations. This manuscript proposes a nuanced perspective that considers the interplay between global and regional changes. By integrating a broader view of cerebral perfusion, we can enhance diagnostic accuracy, uncover new patterns in disease progression, and potentially refine treatment strategies. We advocate for a collaborative effort to validate and implement this comprehensive approach in clinical and research settings.

## Editorial

Arterial spin labeling (ASL) MRI is a non-invasive technique that uses magnetically labeled arterial blood water to measure cerebral blood flow, providing insights into perfusion patterns without the need for contrast agents [1]. It has emerged as a pivotal tool in neuroimaging, providing valuable insights into both regional and global cerebral blood flow changes [1,2]. In the realm of neurodegenerative diseases, ASL has been utilized to identify focal areas of hypoperfusion, corresponding to neuroanatomical regions implicated in specific conditions such as Alzheimer's disease (AD), Parkinson's disease, and dementia with Lewy bodies, among others [1]. These focal changes are now considered an important part of understanding and diagnosing neurodegeneration.

However, this manuscript introduces a proposed perspective suggesting that this regional focus might provide an incomplete picture. Global perfusion alterations, which can affect the entire cerebral landscape, may significantly influence or even mask regional deficits. Conditions such as heart failure, systemic hypertension, or diabetes can induce widespread vascular and blood flow changes, complicating the interpretation of ASL images. This editorial aims to shift the paradigm by advocating for a holistic approach that acknowledges the interplay between global and regional cerebral blood flow changes. By expanding our lens, we can refine diagnostic criteria, monitor disease progression more accurately, and pave the way for more personalized treatment plans.

The current state of ASL in neurodegenerative research

ASL MRI has been instrumental in revealing cerebral blood flow patterns in neurodegenerative diseases. It parallels findings from positron emission tomography and single-photon emission computed tomography in detecting AD-related hypoperfusion in the temporal, parietal, and posterior cingulate cortices. This technique is not only crucial for understanding AD but also predicts transitions from mild cognitive impairment [2]. Similarly, in Parkinson's disease and disorders such as frontotemporal dementia and dementia with Lewy bodies, ASL identifies distinct perfusion abnormalities, assisting in differential diagnosis [1-3]. Its ability to monitor progression over time [4] further underscores its value in neurodegenerative research, enhancing diagnostics and tracking therapeutic impacts. These findings are pivotal in enhancing our understanding of neurodegeneration and in developing diagnostic criteria based on regional blood flow abnormalities.

However, this emphasis on regional deficits, while valuable, might not capture the entire spectrum of cerebral perfusion changes. Neurodegenerative diseases are often accompanied by global vascular alterations that might precede, coincide with, or exacerbate localized pathology. For instance, global reductions in perfusion due to systemic vascular conditions could predispose certain brain regions to more pronounced declines or mask the early stages of neurodegeneration [2]. This raises the question: "Are we seeing the complete picture when we focus solely on regional changes?".

The interplay of global and regional perfusion

**Figure 1:**
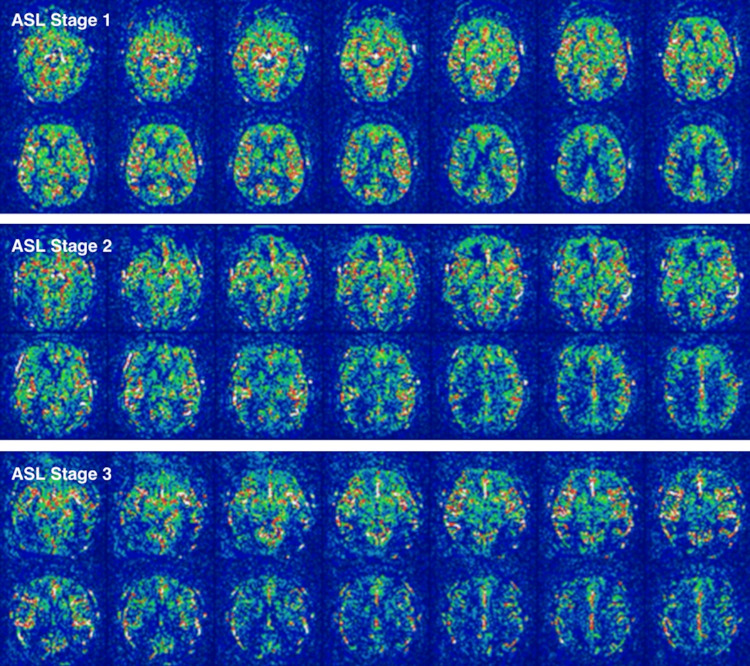
Arterial spin labeling (ASL) perfusion decline ASL perfusion mosaic images demonstrating a stepwise perfusion decline. Areas around the posterior watershed vascular territories are among the first to exhibit changes in the context of widespread perfusion alterations [5].

To address the limitations of a purely regional focus, it is crucial to consider the broader context of cerebral perfusion. A global reduction in perfusion might not only predispose specific regions to more pronounced declines but also complicate the interpretation of seemingly isolated regional deficits. Conversely, what appears to be a localized perfusion deficit might, in reality, be part of a more generalized vascular impairment. This understanding is underscored by the recent development of a global perfusion ASL visual rating scale [5], which offers a qualitative approach to assess the broader cerebral perfusion context before delving into the focal changes (Figure 1). The five-stage ASL visual rating scale evaluates global cerebral perfusion changes, progressing from normal (Stage 0) to severe perfusion decline (Stage 4), with each stage incorporating hallmark features such as reduced perfusion signals and intravascular ASL artifacts. These stages, designed for quick and practical assessment, emphasize defining changes that enable radiologists to systematically interpret perfusion patterns across brain regions [5].

Understanding this interplay is particularly relevant given the multifactorial nature of neurodegenerative diseases. Vascular factors often coexist with and exacerbate primary neuropathological processes [2]. This suggests that a comprehensive evaluation of ASL perfusion should include an assessment of both global and regional changes. Such an approach could offer a more nuanced understanding of neurodegeneration, help differentiate disease-specific perfusion patterns from those related to comorbid conditions or general aging, and potentially lead to more personalized treatment strategies.

Proposing a two-step interpretative approach

In light of these considerations, we propose a two-step interpretative approach for ASL perfusion images in neurodegenerative diseases. The initial step involves establishing a baseline of global cerebral perfusion, which is crucial for a nuanced understanding of each patient's unique perfusion profile [5]. This approach acknowledges the individual variability in cerebral circulation and moves beyond reliance on age-based presumptions or standardized adjustment of post-labeling delay/inversion time. It provides a detailed backdrop against which specific regional abnormalities can be evaluated more accurately. Subsequently, the second step focuses on the analysis of focal changes, ensuring a comprehensive interpretation within this broader context.

Such an approach does not negate the value of identifying regional perfusion deficits but rather enhances the interpretative framework by providing a more holistic view of cerebral perfusion. It acknowledges that both global and regional perfusion changes contribute to the complex landscape of neurodegenerative diseases. This perspective might also facilitate the differentiation of perfusion patterns among various neurodegenerative diseases, which often show overlapping regional deficits but might exhibit distinct global perfusion profiles [1,3].

Conclusion and future directions

Incorporating a global assessment of cerebral perfusion offers a more comprehensive approach to understanding neurodegenerative diseases. This paradigm shift encourages us to consider not just where changes occur in the brain but also how these changes fit within the broader vascular context. As we advance, it is imperative to validate and refine this approach through empirical research, ensuring it is robust, reliable, and applicable across diverse clinical settings.

By integrating global and regional perspectives, we can uncover new patterns and relationships in cerebral blood flow, enhancing the accuracy and reliability of ASL interpretations. This could lead to improved diagnostic criteria, better monitoring of disease progression, and more personalized treatment plans. Collaborative efforts between imaging experts, neurologists, and researchers will be crucial to explore the full potential of this comprehensive approach and ultimately enrich our understanding of neurodegenerative diseases and their impact on cerebral blood flow.
